# Intraoperative incision irrigation with high-volume saline reduces surgical site infection for abdominal infections

**DOI:** 10.3389/fsurg.2022.927896

**Published:** 2022-07-12

**Authors:** Jin Wang, Wen Lv, Shihai Xu, Chao Yang, Bo Du, Yuanbo Zhong, Fei Shi, Aijun Shan

**Affiliations:** Emergency Department, Shenzhen People’s Hospital (The Second Clinical Medical College, Jinan University; The First Affiliated Hospital, Southern University of Science and Technology), Shenzhen, China

**Keywords:** Saline irrigation, surgical site infection, laparotomy, abdominal infection, high-volume

## Abstract

**Purpose:**

Surgical site infection (SSI) remains one of the most common postoperative complications for patients with abdominal infections. This study aimed at investigating the effectiveness of high-volume normal saline (NS) irrigation in preventing postoperative SSI for patients with abdominal infections.

**Methods:**

In this retrospective before-after clinical study, patients who underwent emergency laparotomy due to abdominal infections between Jan 2015 and Dec 2021 were included consecutively. A cohort of 207 patients with NS irrigation was compared to historical controls. A propensity score matching (PSM) with a 1:1 ratio was performed to reduce potential bias. The primary outcome was the 30-day SSI rate.

**Results:**

Irrigation (*n = *207) and control (*n = *207) matched patients were statistically identical on baseline characteristics, perioperative, and intraoperative parameters. Irrigation patients had lower overall SSI rates (10.6% vs. 26.1%, *p* < 0.001), mainly due to reduction in superficial (4.3% vs. 17.9%) and deep (1.4% vs. 3.9%) SSIs, rather than space/organ SSIs (4.8% vs. 4.3%). Irrigation patients also had lower rates of incision seroma (4.8% vs. 11.6%, *p* = 0.012), shorter duration of antibiotics use (5.2 ± 1.7 d vs. 7.2 ± 2.0 d, *p* < 0.001), and unplanned readmission (1.0% vs. 8.7%, *p* < 0.001). Length of hospital stay showed a declining trend with irrigation intervention, while no significant difference was observed. Moreoever, logistic regression revealed that NS irrigationwas an independent protector against SSI (*OR* 0.309; *95% CI,* 0.207–0.462; *p* < 0.001).

**Conclusion:**

Intraoperative incision irrigation with high-volume NS is associated with a lower rate of SSI for patients with abdominal infections.

## Introduction

Surgical site infections (SSI), the most common complications after abdominal surgery, are often observed in clinical practice, varying from 4% to 40% (1, 2). In the emergency setting, SSI contributes to a more significant proportion of postoperative morbidity, with reported rates of 25% to 40% (3, 4). SSI prolongs the hospital length of stay (LOS) and increases the readmission rate and healthcare costs (5). Multiple approaches have been advocated to lower SSI, including adequate bowel preparation, appropriate skin preparation, prophylactic antibiotics, intraoperative maintenance of normothermia, oxygenation, tissue microcirculation perfusion, normoglycemia, use of incision protectors and incision irrigation technique (6, 7). Among these methods, conclusions on incision irrigation remain controversial. Several studies (8–10) showed that incision irrigation reduced the SSI rates. However, a meta-analysis concluded that incision irrigation was not associated with SSI incidence (11). Thus, the role of incision irrigation in preventing SSI warrants further investigation. Normal saline is a common, safe, and inexpensive solution in clinical use. This study aimed to evaluate the efficacy of incision irrigation with high-volume NS on SSI prevention in patients undergoing emergency laparotomy due to abdominal infections.

## Methods

This is a retrospective before-after cohort study conducted in Emergency Center of Shenzhen People's Hospital. Patients who underwent emergency laparotomy for abdominal infections between Jan 2015 and Dec 2021 were enrolled. During the past two years, incision irrigation with high-volume NS began to be gradually performed during laparotomy in order to reduce surgical site infections. Patients admitted before 2020 were defined as a historical control group. Thus, a cohort of 207 patients with NS irrigation (between Jan 2020 and Dec 2021) was compared to historical controls (between Jan 2015 and Dec 2019). Data regarding demographics, admission, perioperative and intraoperative details, and postoperative outcomes were extracted from the Electronic Medical Database of Shenzhen People's Hospital. The Ethics Committee of Shenzhen People's Hospital approved this study and waived written informed consent since only de-identified data were used.

### Inclusion criteria

The inclusion criteria for both irrigation and control groups were identical: Patients with age ≥18 years old undergoing laparotomy for abdominal infections were included. The exclusion criteria were as follows: laparoscopic operations; trauma patients; pregnancy; follow-up information missing.

### Irrigation intervention

All surgical procedures were performed under general or spinal anesthesia, depending on anesthesiologists. After skin sterilization with 0.5% povidone-iodine solution, the surgical area was laid with sterile towels and covered with a labeled surgical incision protective film. Intraoperative procedures were determined on a case-by-case basis.

In the irrigation group, after the closure of the peritoneum, high-volume NS (generally 50–200 ml NS per 1 cm incision, at least 500 ml, total volume 500–5000 ml, depending on the incision length) was used to irrigate the abdominal muscle and the subcutaneous soft tissue around the incisions. The whole incision surface was carefully rinsed with gauze scrub layer-by-layer in an attempt to eliminate bacteria, tissue debris, and blood clots. The first irrigation was performed after peritoneum closure; the second irrigation was conducted after the suture of the fascia. Detailed surgical procedures are shown in the Supplementary video file. For the control group, standard surgical procedures were performed without NS irrigation. Patients in both groups identically received primary closure for incisions.

### Outcome assessment

The primary outcome was 30-day SSI, complying with the CDC criteria (12), in which SSI was defined as an infection that occurs within 30 days postoperatively meanwhile the patient at least has one of the following: purulent drainage from the incision, positive cultures of the incision swab, incision reopened spontaneously or by a clinician, localized erythema/swelling/heat/pain/tenderness, systemic fever (>38°C), diagnosis of SSI from a clinician or radiological imaging. Secondary outcomes included incision dehiscence, incision hematoma, incision seroma, duration of antibiotics use, unplanned readmission, and unplanned reoperation. Hospital length of stay and 30-day mortality were also assessed. After discharge, patients were routinely requested to get back for follow-up through day 30.

### Statistical analysis

To reduce the potential bias from confounding factors between groups, propensity-score matching (PSM) was performed with a 1:1 ratio using the nearest-neighbor method without replacement. PSM was used to adjust eleven covariates, which included age, gender, body mass index (BMI), comorbidity, American Society of Anesthesiologists (ASA) score, white blood cell (WBC), procalcitonin (PCT), onset-to-operation time, surgical indication, incision length, and incision classification.

The irrigation and control group were compared with respect to perioperative characteristics and postoperative outcomes. Continuous variables were described using mean ± SD or median (IQR) (if skewed) and were analyzed with independent samples *t*-tests or Wilcoxon rank-sum test. Categorical variables were summarized as number (percentage) and were compared with chi-squared test or Fisher exact test where appropriate. Binary logistic regression was performed to determine the independent predictors of SSI *via* the comparison between SSI-positive patients and SSI-negative patients. Odds ratios (*OR*) with their respective 95% confidence intervals (*95% CI*) were used to assess for statistical associations.

For subgroup analyses, a series of baseline characteristics, including gender, age, BMI, COPD, hypertension, diabetes, CHD, ASA, PCT, surgical indication, incision length, and incision classification, were selected for stratification, in which age, BMI, PCT, and incision length were transformed into dichotomous variables. *Cutoff* values for separating PCT and incision length were defined as median values. Binary logistic regression analysis was performed to evaluate the *OR* of SSI within each subgroup, and results were visualized by the forest function provided in the R-package of meta. *p*-value <0.05 was considered statistically significant. All statistical analyses were two-sided and were performed with R language 3.6.2 version (R Foundation).

## Results

### Baseline characteristics

**Figure 1:**
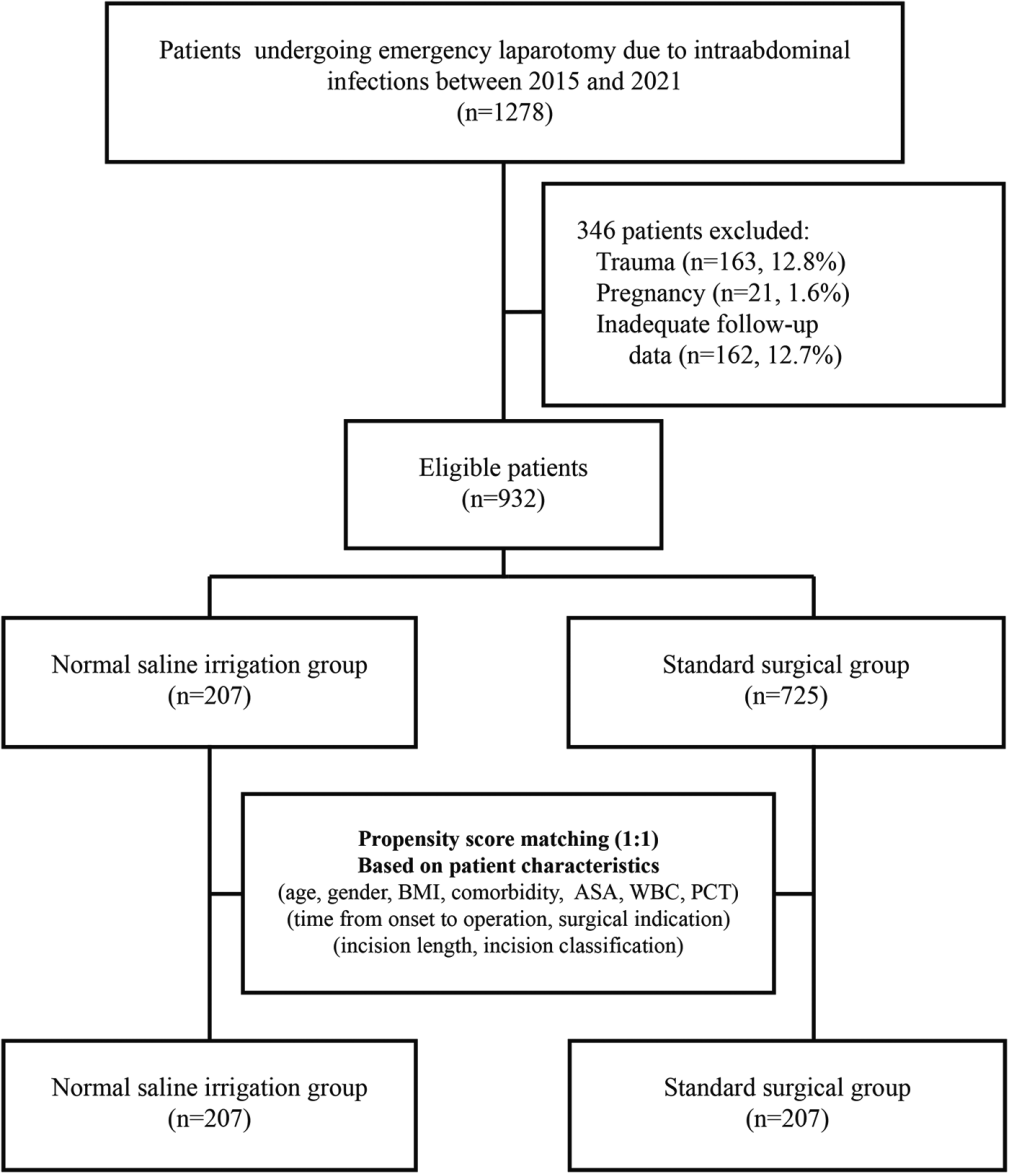
Flowchart of the study.

A total of 1278 patients undergoing emergency laparotomy for abdominal infections were reviewed (Figure 1). Three hundred and forty-six patients were excluded because they did not fulfil the eligibility criteria. Overall, 207 patients received incision irrigation. Supplementary Table S1 compares the baseline characteristics between irrigation and non-irrigation in the unmatched cohort. Patients’ baseline characteristics, including age, gender, BMI, ASA, the majority of comorbidities (regarding COPD, diabetes, dialysis, coronary heart disease, cancer, cirrhosis, and stroke), WBC, PCT, surgical indication, incision length, and incision classification, were similar. While irrigation patients had a higher percentage of hypertension (30.4% and 22.9%, *p* = 0.026), steroid use (6.3% and 2.3%, *p* = 0.005), and prolonged onset-operation time (median 21.8 h vs. 20.0 h, *p* = 0.007) in the unmatched cohorts. After PSM matching, patients' characteristics between irrigation (*n* = 207) and control (*n* = 207) groups were all similar, with the preexisting differences being well balanced (Table 1).

**Table 1:** Baseline characteristics after propensity score matching.

Characteristics	Control group (*n* = 207)	Irrigation group (*n* = 207)	*p-*value
Gender, male, *n* (%)	136(65.7)	131(63.3)	0.608
Age, mean ± SD, y	55.6 ± 8.0	55.3 ± 8.0	0.754
BMI, mean ± SD, kg/m^2^	21.7 ± 3.0	21.9 ± 3.1	0.546
Comorbidities, *n* (%)			
Severe COPD	23(11.1)	22(10.6)	0.875
Hypertension	64(30.9)	63(30.4)	0.915
Diabetes	35(16.9)	34(16.4)	0.895
Dialysis	3(1.4)	6(2.9)	0.312
Coronary heart disease	20(9.7)	23(11.1)	0.629
Cancer	9(4.3)	8(3.9)	0.804
Steroid use	12(5.8)	13(6.3)	0.837
Cirrhosis	8(3.9)	12(5.8)	0.359
Stroke	9(4.3)	7(3.4)	0.610
ASA, *n* (%)			0.708
1	66(31.9)	68(32.9)	
2	93(44.9)	88(42.5)	
3	28(13.5)	35(16.9)	
4	20(9.7)	16(7.7)	
WBC, mean ± SD, ×10^9^/l	15.3 ± 4.0	14.6 ± 4.0	0.083
PCT, median and IQR, ng/ml	5.3(2.6–7.9)	5.7(2.2–8.2)	0.525
Onset to operation time, median and IQR, h	22.1(16.6–27.8)	21.8(15.3–30.3)	0.813
Surgical indication, *n* (%)			0.938
Appendicitis	63(30.4)	67(32.4)	
Diverticulitis	21(10.1)	23(11.1)	
Upper gastrointestinal perforation	54(26.1)	57(27.5)	
Small bowel perforation	23(11.1)	23(11.1)	
Pancreatitis	13(6.3)	11(5.3)	
Colorectal perforation	22(10.6)	15(7.2)	
Primary intraabdominal infection	11(5.3)	11(5.3)	
Incision length, mean ± SD, cm	4.7 ± 1.5	4.6 ± 1.7	0.441
Incision, *n* (%)			0.960
Clean/contaminated	24(11.6)	25(12.1)	
Contaminated	147(71.0)	148(71.5)	
Dirty	36(17.4)	34(16.4)	

*Bold values mean Statistically significant.*

### Outcomes

Irrigation patients had lower overall SSI rates (10.6% vs. 26.1%, *p* < 0.001), mainly due to reduction in superficial (4.3% vs. 17.9%) and deep (1.4% vs. 3.9%) SSIs, rather than space/organ SSIs (4.8% vs. 4.3%). Moreover, irrigation was associated with a lower rate of incision seroma (4.8% vs. 11.6%, *p =* 0.012), shorter duration of antibiotics use (5.2 ± 1.7 d vs. 7.2 ± 2.0 d, *p <* 0.001), lower rate of unplanned readmission (1.0% vs. 8.7%, *p* < 0.001). Length of hospital stay showed a declining trend with irrigation intervention, while no significant difference was observed (10.5 ± 6.6 d vs. 11.4 ± 6.2 d, *p* = 0.155). There were no overall differences in incision dehiscence, incision hematoma, unplanned reoperation, and 30-day mortality between the two groups (Table 2).

**Table 2:** Postoperative outcomes within 30 days.

Outcome	Control group (*n *= 207)	Irrigation group (*n *= 207)	*p-*value
Primary outcome			
Surgical site infection, *n* (%)	54(26.1)	22(10.6)	**<0**.**001**
Superficial SSI	37(17.9)	9(4.3)	
Deep SSI	8(3.9)	3(1.4)	
Organ space	9(4.3)	10(4.8)	
Secondary outcomes			
Wound dehiscence, *n* (%)	20(9.7)	18(8.7)	0.734
Wound hematoma, *n* (%)	4(1.9)	4(1.9)	1.000
Wound seroma, *n* (%)	24(11.6)	10(4.8)	**0**.**012**
Duration of antibiotics use, mean ± SD, d	7.2 ± 2.0	5.2 ± 1.7	**<0**.**001**
Unplanned readmission, *n* (%)	18(8.7)	2(1.0)	**<0**.**001**
Unplanned reoperation, *n*(%)	10(4.8)	4(1.9)	0.103
Length of stay, mean ± SD, d	11.4 ± 6.2	10.5 ± 6.6	0.155
Mortality, *n*(%)	4(1.9)	6(2.9)	0.522

*Bold values mean Statistically significant.*

### Subgroup analysis of SSI

**Figure 2:**
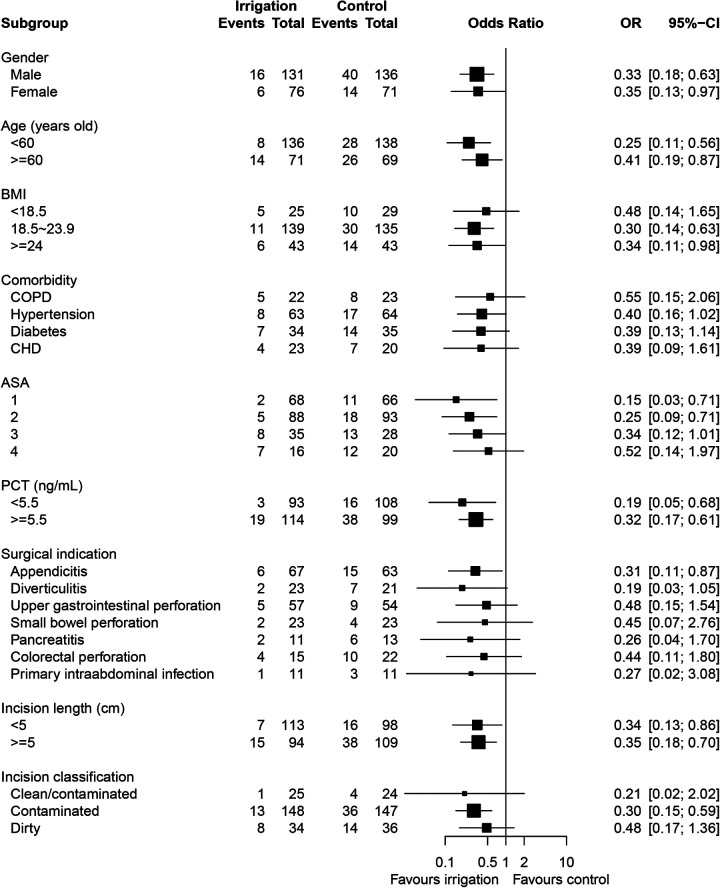
Subgroup analysis for surgical site infection in the irrigation group as compared with the control group, assessed by logistic regression analysis with adjustment for the stratification variables.

To further explore the potential heterogeneity in reducing SSI, subgroup analyses were performed across different baseline characteristics. As shown in Figure 2, no heterogeneity was observed in the effect of irrigation group vs. control group on SSI with regard to different levels of gender, age, PCT, and incision length. We observed heterogeneity in the effect evaluation of irrigation among patients with different levels of BMI, comorbidity, ASA, surgical indication, as well as incision classification. Generally, patients with relatively better physical health and fewer underlying diseases seemed to have a higher response rate to irrigation intervention. However, despite no significance in some subgroups, there also showed an apparent trend in SSI reduction for patients undergoing irrigation**,** suggesting the potential effectiveness of irrigation across different patients.

### Predictors of SSI

Within the PSM-matched cohort, univariate logistic regression revealed that variables including NS irrigation, age, PCT, and surgical indications were significantly correlated with SSI occurrence. While the multivariate regression model suggested that NS irrigation (*OR* 0.309; *95% CI,* 0.207–0.462; *p* < 0.001), PCT, and surgical indications were independent predictors of SSI, with the variable of age excluded from the model (Table 3).

**Table 3:** Logistic regression analysis for surgical site infection.

	Univariate		Multivariate	
	*OR (95% CI)*	*p-*value	*OR (95% CI)*	*p-*value
NS irrigation	0.337 (0.193–0.571)	0.000	0.309 (0.207–0.462)	**0**.**000**
Age	1.044 (1.012–1.079)	0.008	1.013 (0.981–1.046)	0.431
PCT	1.187 (1.013–1.391)	0.034	1.012 (1.003–1.021)	**0**.**009**
Surgical indication				
Upper gastrointestinal perforation	1		1	** **
Small bowel perforation	1.090(0.963–1.233)	0.173	1.083(0.965–1.216)	0.175
Appendicitis	1.292(0.971–1.719)	0.079	1.289(0.968–1.715)	0.081
Primary intraabdominal infection	1.492(0.943–2.362)	0.087	1.895(1.216–2.953)	**0**.**005**
Diverticulitis	1.798(1.214–2.662)	0.003	1.896(1.337–2.682)	**0**.**000**
Pancreatitis	3.203(2.311–4.439)	0.000	3.301(2.263–4.816)	**0**.**000**
Colorectal perforation	4.218(3.314–5.368)	0.000	4.321(3.324–5.617)	**0**.**000**

## Discussion

This study confirmed that incision irrigation with NS during emergency laparotomy significantly reduces SSI rates for patients with abdominal infections. Subsequent subgroup analyses validated the potential effectiveness of NS irrigation among patients with different baseline characteristics. The logistic regression analysis revealed that NS irrigation played an independently protective role against SSI. Furthermore, NS irrigation lowered incision seroma rates. Consequently, the duration of antibiotics use was shortened, and the readmission rate was decreased. Despite no difference in LOS change, there also showed an apparent trend that the LOS was shortened from an average of 11.4 days to 10.5 days. These results suggested that incision irrigation with high-volume NS could effectively prevent SSI and incision seroma during emergency laparotomy for abdominal infections.

Surgical site infection is one of the most prevalent complications after laparotomy. SSI results in delayed rehabilitation, long-term disability, and later risk for incisional hernia (13). The causes of SSI are multifactorial, deriving from the interplay among surgical, environmental, and patient-related factors (14). Wound contamination of bacteria is considered the most common factor, followed by deposition of tissue debris and blood clots, especially during laparotomy (15). Thus, theoretically, irrigation flushes out bacteria, blood clots and tissue debris from the incision, which are the most critical factors for developing SSI. Intraoperative incision irrigation with high-volume NS is a safe, feasible, and inexpensive technique. Nevertheless, current conclusions on NS irrigation remain controversial, and evidence is insufficient. Despite multiple types of solutions being explored for incision irrigation, studies specifically designed for NS irrigation are scarce. Only five studies explored NS irrigation in abdominal operations while reporting contradictory results. The earliest study (16) performed by Cervantes showed that SSI was reduced from 25% to 8.7% with a volume of 300 ml NS irrigation in appendectomy patients. However, three other studies from obstetrics and gynaecology consistently reported no association between NS irrigation and SSI rate. Al-Ramahi et al. (17) reported NS irrigation did not reduce the SSI rate during gynaecological surgery (10.6% irrigation vs. 9.8% control). Gungorduk et al. (18) concluded NS irrigation was not associated with SSI rates for caesarean section (6.5% irrigation vs. 7.3% control, *p* = 0.86). Aslan et al. (19) also revealed the limited effect of NS irrigation on SSI reduction for cesarean section (14.3% irrigation vs. 12.8% control). Recently, Emile (20) performed a randomized trial to compare gentamycin-saline, saline-only vs. no-irrigation in appendectomy patients, and the results demonstrated a powerful effect of NS irrigation on SSI prevention (4.3% gentamycin-saline, 2.9% saline-only, and 17.4% no-irrigation). Our results support the application of NS irrigation in abdominal operations, consistent with conclusions from Cervantes and Emile.

Regarding reasons for the inconsistent conclusions, we speculate that it may be associated with the different volumes of NS across these studies. Al-Ramahi, Gungorduk and Aslan used 50, 100 and 200 ml of NS for incision irrigation, respectively, in their gynaecological and obstetric surgery patients. However, Cervantes and Emile used a large volume of NS (300 and 400 ml, respectively) for appendectomy patients. Similarly, we prefer high-volume NS for incision irrigation (see Method section). Gynaecological and obstetric operations usually create incisions more than 10 cm, while appendectomies only require 3–5 cm incisions. Therefore, high-volume NS irrigation in large incisions may function. With the increase in the volume of irrigation, the decline in bacterial load is supposed to be logarithmic.

NS irrigation also lowered incision seroma rates, which further confirmed its effectiveness in protecting incisions against multiple complications. Wound seroma is a collection of serous fluid accumulated in the soft tissue dead-space, in most cases deriving from inflammatory exudate, adipose tissue liquefaction, and leakage of lymph fluid (14). Seroma, as a potential prodromal symptom of SSI, can be observed in 3%–10% of patients undergoing laparotomy (14). NS irrigation effectively evacuates blood clots, dead cells, and matrix debris from the wound, and thereby preventing seroma formation. Consistent results were also reported by Aslan in their report on cesarean sections (19). Moreover, the unplanned readmission reflects the severity of incision complications. In the irrigation cohort, a reduction in the readmission rate suggested that irrigation not only decreased the SSI rates but also alleviated the severity of incision complications.

The key strength of our study is using a series of pragmatic and real-world data consisting of heterogenous emergency patients. Propensity score matching was performed to reduce the potential bias between groups. Our study adds new evidence on the efficacy of high-volume NS irrigation during laparotomy, which may contribute to finding a simple, safe and inexpensive technique for SSI prevention. However, limitations still exist in our work, mainly focusing on the retrospective before-after characteristics. Despite no substantial alterations regarding perioperative management, anaesthesia technique, and intraoperative procedures during the study period, clinical conditions might have possibly changed besides the intervention factor, particularly in the improvement of the clinical experience of our medical care team. An additional limitation was that excluding patients with missing follow-up information might generate potential bias.

## Conclusions

Intraoperative incision irrigation with high-volume NS significantly reduces the rate of SSI and seroma for patients undergoing laparotomy due to abdominal infections compared to no-irrigation, thereby contributing to a reduction in the duration of antibiotic use and unplanned readmission. Moreover, high-volume NS irrigation is a feasible and inexpensive approach for preventing SSI, warranting clinical application across different medical institutions.

## Data Availability

The raw data supporting the conclusions of this article will be made available by the authors, without undue reservation.
