# Supplementary material for: Intraoperative incision irrigation with high-volume saline reduces surgical site infection for abdominal infections
Source: Front Surg. 2022 Jul 12;9:927896. doi: 10.3389/fsurg.2022.927896 (PMC9314743; doi:10.3389/fsurg.2022.927896)
Supplement: Supplementary file 1 [file Table_1_v1.docx]

**Supplemental table 1. Baseline characteristics before propensity matching**

| Characteristics | Control group  (n=725) | Irrigation group  (n=207) | *P* value |
| --- | --- | --- | --- |
| Gender, male, n (%) | 409(56.4) | 131(63.3) | 0.077 |
| Age, mean ± SD, y | 56.0±8.2 | 55.3±8.0 | 0.281 |
| BMI, mean ± SD, kg/m^2^ | 22.0±3.0 | 21.9±3.1 | 0.702 |
| Comorbidities, n (%) |  |  |  |
| Severe COPD | 65(9.0) | 22(10.6) | 0.468 |
| Hypertension | 166(22.9) | 63(30.4) | **0.026** |
| Diabetes | 129(17.8) | 34(16.4) | 0.648 |
| Dialysis | 23(3.2) | 6(2.9) | 0.841 |
| Coronary heart disease | 83(11.4) | 23(11.1) | 0.893 |
| Cancer | 24(3.3) | 8(3.9) | 0.699 |
| Steroid use | 17(2.3) | 13(6.3) | **0.005** |
| Cirrhosis | 28(3.9) | 12(5.8) | 0.226 |
| Stroke | 30(4.1) | 7(3.4) | 0.623 |
| ASA, n (%) |  |  | 0.420 |
| 1 | 250(34.5) | 68(32.9) |  |
| 2 | 274(37.8) | 88(42.5) |  |
| 3 | 151(20.8) | 35(16.9) |  |
| 4 | 46(6.3) | 16(7.7) |  |
| 5 | 4(0.6) | 0(0.0) |  |
| WBC, mean ± SD, ×10^9^/L | 14.7±4.0 | 14.6±4.0 | 0.715 |
| PCT, median and IQR, ng/mL | 5.03(2.2-8.0) | 5.71(2.2-8.2) | 0.374 |
| Onset to operation time, median and IQR, h | 20.0(13.5-26.8) | 21.8(15.3-30.3) | **0.007** |
| Surgical indication, n (%) |  |  | 0.599 |
| Appendicitis | 207(28.6) | 67(32.4) |  |
| Diverticulitis | 91(12.6) | 23(11.1) |  |
| Upper gastrointestinal perforation | 180(24.8) | 57(27.5) |  |
| Small bowel perforation | 107(14.8) | 23(11.1) |  |
| Pancreatitis | 34(4.7) | 11(5.3) |  |
| Colorectal perforation | 72(9.9) | 15(7.2) |  |
| Primary intraabdominal infection | 34(4.7) | 11(5.3) |  |
| Incision length, mean ± SD, cm | 4.6±1.5 | 4.6±1.7 | 0.782 |
| Incision classification, n (%) |  |  | 0.559 |
| Clean/contaminated | 109(15.0) | 25(12.1) |  |
| Contaminated | 498(68.7) | 148(71.5) |  |
| Dirty | 118(16.3) | 34(16.4) |  |
